# Effects of corticosteroid on the expressions of neuropeptide and cytokine mRNA and on tenocyte viability in lateral epicondylitis

**DOI:** 10.1186/1476-9255-9-40

**Published:** 2012-10-30

**Authors:** Soo Hong Han, Hee Jung An, Ji Ye Song, Dong Eun Shin, Young Do Kwon, Jong Sup Shim, Soon Chul Lee

**Affiliations:** 1 Department of Orthopaedic Surgery, CHA Bundang Medical Center, CHA University, Gyeonggi-do, 463-712, Korea; 2 Department of Pathology, CHA Bundang Medical Center, CHA University, Gyeonggi-do, 463-712, Korea; 3 Institute for Clinical Research, CHA Bundang Medical Center, CHA University, Gyeonggi-do, 463-712, Korea; 4 Department of Biomedical Science, Graduate School, CHA University, Gyeonggi-do, 463-712, Korea; 5 Department of Orthopaedic Surgery, Samsung Medical Center, Sungkyunkwan University, Seoul, 135-710, Korea

**Keywords:** Lateral epicondylitis, Corticosteroid, Neuropeptide, mRNA, Tenocyte viability

## Abstract

**Background:**

The purpose of this study was to determine the reaction mechanism of corticosteroid by analyzing the expression patterns of neuropeptides (substance P (SP), calcitonin gene related peptide (CGRP)) and of cytokines (interleukin (IL)-1α, tumor growth factor (TGF)-β) after corticosteroid treatment in lateral epicondylitis. In addition, we also investigated whether corticosteroid influenced tenocyte viability.

**Methods:**

The corticosteroid triamcinolone acetonide (TAA) was applied to cultured tenocytes of lateral epicondylitis, and the changes in the mRNA expressions of neuropeptides and cytokines and tenocyte viabilities were analyzed at seven time points. Quantitative real-time polymerase chain reaction and an MTT assay were used.

**Results:**

The expression of SP mRNA was maximally inhibited by TAA at 24 hours but recovered at 72 hours, and the expressions of CGRP mRNA and IL-1α mRNA were inhibited at 24 and 3 hours, respectively. The expression of TGF-β mRNA was not significant. Tenocyte viability was significantly reduced by TAA at 24 hours.

**Conclusions:**

We postulate that the reaction mechanism predominantly responsible for symptomatic relief after a corticosteroid injection involves the inhibitions of neuropeptides and cytokines, such as, CGRP and IL-1α. However the tenocyte viability was compromised by a corticosteroid.

## Background

Lateral epicondylitis is histologically characterized by angiofibroblastic hyperplasia, which includes hypercellularity, neovascularization, increased protein synthesis, and disorganization of the matrix, but not inflammation [1-6].

However, though many authors have suggested different, such as, abnormal vascularity, [7] an imbalance between vasoconstrictor and vasodilator innervations [8] micro-tears, [9] and degenerative changes, [10] the pathogenesis of lateral epicondylitis remains obscure. Recent studies have suggested that neuropeptides, such as, substance P (SP) and calcitonin gene related peptide (CGRP), are involved in its pathogenesis [8,11,12] because inflammatory responses, such as, increased vascular permeability and edematous changes are evident, though inflammatory cells are absent.

Ljung BO et al. [11] analyzed six patients suffering from lateral epicondylitis and six healthy volunteers using immunohistochemistry, and suggested that nerve fibers showing SP-like and CGRP-like immunoreactivity were demonstrated at the origin of the extensor carpi radialis brevis muscle, and neurogenic inflammation might be implicated in the etiology of lateral epicondylitis. Uchio Y et al. [8] also concluded that neuropeptides (SP, CGRP) and cytokines (interleukin (IL)-1α, tumor growth factor (TGF)-β) might contribute to the pathology of lateral epicondylitis, and suggested that further studies would be conducted to clarify the interactions between neuropeptides and cytokines. However, this study was conducted on only 9 patients, and the analysis was based on immunohistochemistry determined protein levels.

Corticosteroid injection is often used to treat lateral epicondylitis, and high short-term success rates have been reported [13]. However, long-term efficacy is controversial with high rates of recurrence [14,15]. Furthermore, several reports of tendon weakness or spontaneous rupture after corticosteroid injection have been issued [16].

Before experiment, because corticosteroids are strong anti-inflammatory agents, we speculated that they might have a regulatory effect on neuropeptides and cytokines in the lateral epicondylitis. Furthermore based on the previously reported studies, we assumed that the mRNA expression of neuropeptides and cytokines in the tenocyte of lateral epicondylitis would be increased in vitro.

Then we analyzed the mRNA expression patterns of neuropeptides and cytokines using quantitative real-time polymerase chain reaction (PCR) after treatment with corticosteroid (triamcinolone acetonide) to determine the reaction mechanism responsible for the short-term symptomatic relief afforded by corticosteroid in lateral epicondylitis. In addition, we examined tenocyte viability to investigate the detrimental effects of corticosteroid on tendon structure using a 3-(4,5-dimethylthiazol-2-yl)-2,5-diphenyl-tetrazolium bromide (MTT) assay.

## Methods

### Tissue Sampling

#### Diseased tendon

The institutional review board at CHA Bundang hospital approved this study. After written informed consent was obtained, twenty-seven patients (11 men and 16 women; mean 47.5 (SD ± 9.47 years)) treated during the period 2008 - 2012 were studied. All patients underwent partial resection of the extensor carpi radialis brevis origin with drilling of the lateral condyle for lateral epicondylitis because nonoperative treatment had failed. Twenty patients had lateral epicondylitis on the dominant side, and seven on the nondominant side. Before operation, all patients were treated nonoperatively, this included; cessation of the offending activity, bracing, physiotherapy (ultrasound, electrical stimulation, manipulation, stretching, and strengthening exercises), and anti-inflammatory agents. Twenty of the 27 were given a local injection of corticosteroid (numbers of injections ranged from 1 to 3; mean 1.59). No patients received extracorporeal shock wave therapy or platelet-rich plasma or botulinum toxin A injection before surgery. No patient was injected with platelet-rich plasma or botulinum toxin A.

Diagnoses were made by history taking and based on physical exam findings, including tenderness over the tendinous origin close to the lateral epicondyle. Pain was evoked by resisted extension of the wrist joint, and symptom duration ranged from 3 to 34 months (mean duration 18.6 months). Alternative diagnoses, such as, synovitis of the proximal radioulnar joint, entrapment of the radial nerve, compartment syndrome in anconeus muscle, arthritis, and other joint disease, were excluded by clinical examination. In addition, patients with a history of surgery involving the elbow joint were excluded.

Under general or regional anesthesia, an open approach (a modification of the Nirschl procedure) was used, which involved the release of extensor muscle tendon with decortication at their origin on the lateral. During each operation, biopsy specimens (7-9 × 7-9 × 5 mm^3^) were taken from the extensor carpi radialis brevis tendon, which appeared dull, gray, friable, and edematous in all 27 patients. One senior surgeon performed the operations and biopsies.

#### Healthy tendon

For the control study, we harvested the tendon of extensor carpi radialis brevis from healthy donor, and used it as the control group. Among the 5 healthy tendons, three was harvested from men and 2 from women (mean 45.4 (SD ± 9.47 years)). Then peliminarily we extracted the RNA and compared the expressions of neuropeptide and cytokine to that of the diseased tendon before cell culture. The detail method of the RNA extraction is described in the below.

### Preliminary study: comparsion of expressions of neuropeptides and cytokines between the healthy tenocyte and the diseased tenocyte

**Figure 1:**
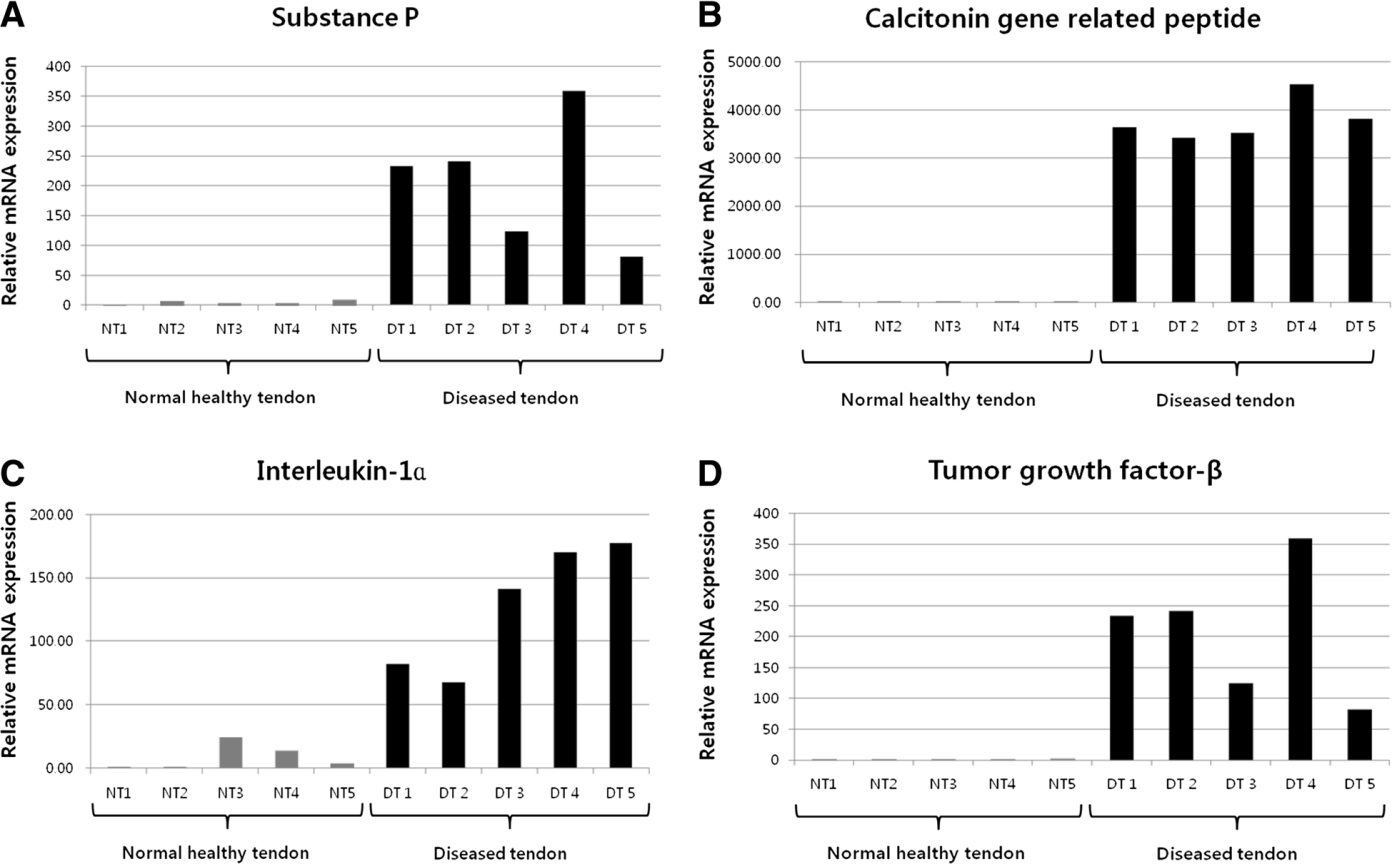
**Results of quantitative real time polymerase chain reaction of neuropeptide and cytokine between the healthy tenocyte and the diseased tenocyte.** (**A**) substance P (**B**) calcitonin gene related peptide (**C**) interleukin-1α (**D**) tumor growth factor-β.

We found the relative expressions of SP, CGRP, IL-1α, and TGF-β in healthy control were extremely limited compared to the expressions in diseased group (Figure 1). The relative mRNA expression of SP in diseased tendon was 51.6 times higher compared to the expression in a control, CGRP 697.0 times, IL-1α 14.7 times, and TGF-β 179.3 times.

### Cell culture

Biopsy tissues were rinsed once in phosphate-buffered saline (PBS), cut into small pieces under sterile conditions, and centrifuged for 5 minutes at 1000 rpm. Pellets were digested for 5 hours in Dulbecco's modified Eagle's medium (DMEM) supplemented with 10 mg/ml CollagenaseII (Gibco, Carlsbad, CA, USA) at 37°C in 5% CO_2_ at 95% relative humidity (RH), CollagenaseII was filtered using a MILLEXGP Filter Unit (Millipore, Bedford, MA, USA). After digestion, CollagenaseII was deactivated by adding fetal bovine serum and the mixture was centrifuged for five minutes at 1000 rpm. The cells obtained were rinsed with PBS, seeded into a 100 π dish and cultured with DMEM supplemented with 10% FBS and 1% Antibiotic-Antimycotic solution (Gibco, Carlsbad, CA, USA) for one week. The culture medium was changed every two days. Tenocytes were cultured until passage 5 and treated with corticosteroid.

### Corticosteroid (triamcinolone acetonide) treatment

Tenocytes were treated with 100 mM triamcinolone acetonide (TAA) (Dongkwang, Seoul, Korea). After mixing TAA 1 vial (40mg/ml) in 10ml DMEM and 1 ml normal saline, it was voltexing sufficiently. For incubation, the mixture 10 ml treated in dish of tendon cell immediately. At baseline 0 (no TAA treatment), and after 1, 3, 5, 24, 48, or 72 hours of treatment, cells were washed with PBS, treated with trypsin-EDTA, and incubated at 37°C in 5% CO2 at 95% RH for five minutes. Tenocytes were subsequently collected by centrifugation at 1000 rpm for 5 minutes.

### RNA isolation and quantitative real-time PCR

RNA was extracted using an RNeasy Mini Kit (Qiagen, GmbH, Hilden, German) and quantified using a NanoDrop Spectrophotometer (NanoDrop Technologies, USA). First-strand cDNA synthesis was performed using a Superscript III kit (Invitrogen, Carlsbad, CA, USA). cDNA samples were analyzed in triplicate using the Bio-Rad CFX96 Real-Time PCR Detection System. Briefly, 1 μg of total RNA was amplified using the Taq Man Gene Expression Assay (Applied Biosystems, UK) for the analyses of GAPDH (ABI code: Hs99999905_m1), SP (ABI code: Hs00243225_m1), CGRP (ABI code: Hs00265194_m1), IL-1α (ABI code: Hs00174092_m1), and TGF-β (ABI code: Hs00998133_m1) respectively. The PCR reaction mix had a volume of 20 μl and contained 10 μl 2X TaqMan master mix (Applied Biosystems, UK), 1 ul primer and probe kit (Applied Biosystems, UK), 1 μl cDNA, and 8 μl of diethylenepyrocarbonate (DEPC) water. The reverse transcription conditions used were as follows; 50°C for 2 minutes, 95°C for 10 minutes, followed by 40 cycles of 95°C for 15 seconds and 60°C for 1 minute. RNA levels were quantified at least three times. Transcript levels were normalized versus GAPDH expression, and gene expression was calculated using 2^-ΔΔCt^.

### MTT assay

Cell viabilities were measured using an MTT assay. The cells were incubated with TAA for 0 (baseline), 1, 3, 5, 24, 48, or 72 hours. Tendon cells were seeded into 96-well plates at a density of 5 × 10^3^ cells per well and incubated at 37°C and 95% RH in 5% CO_2_. After incubation, 10 μl of MTT (Sigma, St. Louis, MO) was added to each well, and plates were incubated for 3 hours at 37°C in 5% CO2. The 100 μl DMSO (Sigma Aldrich, St. Louis, MO) was then added and agitated for 30 minutes. The optical densities were measured using an ELISA reader (Molecular Devices Emax) at an excitation wavelength of 530 nm and an emission wavelength of 590 nm. Readings were taken at least three times at all times for each patient.

### Statistical analysis

Data were collected and analyzed using R software (version 2.11.1; R Foundation for Statistical Computing, Vienna, Austria). To analyze the time-dependent effects of TAA, repeated measures ANOVA was used to determine whether the relative mRNA expressions and optical densities were significantly different at the seven time points. To analyze quantitative real-time PCR results, we arbitrarily set mRNA expression at baseline to one. In addition, we assessed the relationships between the five variables, that is, the relative expression levels of the mRNAs of SP, CGRP, IL-1α, and TGF-β, and tenocyte viability at each time point by Pearson's correlation analysis, and calculated Pearson's correlation coefficients (r). Differences were considered statistically significant when *P* values were less than 0.05 (*p* < 0.05).

## Results

All of the statistical analyses at each time point followed a normal distribution and are summarized in Table 1.

**Table 1:** Changes in gene expression of neuropeptides, cytokines and in optical density of tenocyte viability after triamcinolone acetonide treatment at seven time points (0, 1, 3, 5, 24, 48, 72 hours)

	**No TAA**	**After TAA treatment**						Statistical significance (*p*)*
	**0 hr**	**1 hrs**	**3 hrs**	**5 hrs**	**24 hrs**	**48 hrs**	**72 hrs**	
**SP**	1.000	0.527± 0.154	0.881 ±0.326	0.652 ±0.246	0.412±0.166	0.527 ±0.189	0.696 ±0.235	0.0078
**CGRP**	1.000	1.119± 0.336	1.197 ±0.308	0.958 ±0.312	0.359 ±0.153	0.268 ±0.161	0.330 ±0.164	< 0.0001
**IL-1α**	1.000	1.183 ±0.35	0.342 ±0.168	0.185± 0.091	0.210 ±0.166	0.107± 0.114	0.053 ±0.027	< 0.0001
**TGF-β**	1.000	1.108 ±0.225	1.132 ±0.219	1.186±0.249	1.015±0.198	0.859 ±0.234	0.772 ±0.228	0.0187
**Tenocyte viability**	0.582	0.491± 0.038	0.502 ±0.041	0.467 ±0.049	0.405 ±0.042	0.353 ±0.033	0.306 ±0.021	< 0.0001

The values are given either as mean ± SD.

SP = Substance P.

CGRP = Calcitonin gene related peptide.

IL-1α = Interleukin-1α.

TGF-β = Tumor growth factor-β.

TAA = Triamcinolone acetonide.

*Repeated measures ANOVA.

### Effects of TAA on the expressions of the mRNAs of Neuropeptides (Figure 2)

**Figure 2:**
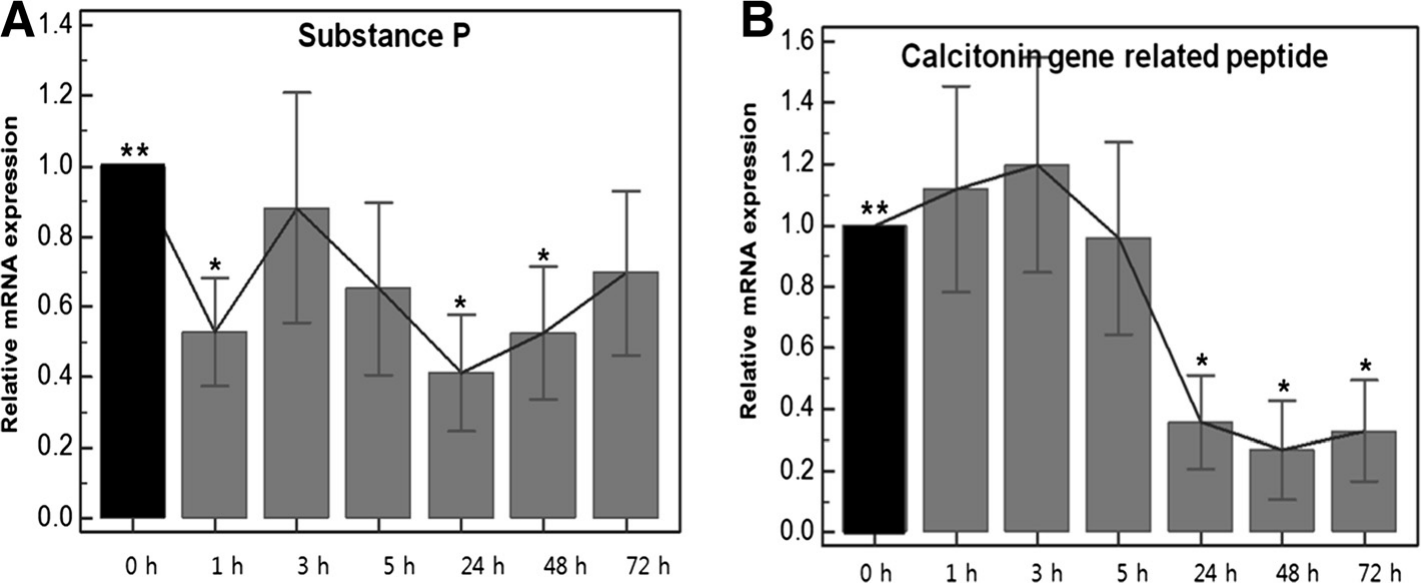
**Results of quantitative real time polymerase chain reaction of neuropeptide after triamcinolone acetonide treatment (0, 1, 3, 5, 24, 48, 72 hours).** (**A**) Changes in mRNA expressions of substance P (**B**) Changes in mRNA expressions of calcitonin gene related peptide **Baseline *Statistically significant values compared to baseline.

#### SP

The SP mRNA expression differed between the seven time points of the protocol (*p* = 0.0078, repeated measures ANOVA), and its expression was substantially decreased after 1, 24, and 48 hours of TAA treatment versus baseline (*p* < 0.0001, < 0.0001, = 0.0005, each), but followed by a minimal increase from 48 to 72 hours. However, values at 3 and 5 hours were not significantly different from baseline.

#### CGRP

Repeated measures ANOVA revealed that CGRP expression differed at the seven treatment times (*p <* 0.0001). The mRNA expression started to reduce substantially after 24 hours of TAA treatment. The gene expression of CGRP at 24, 48, and 72 hours was significantly diminished by 64.1% at 24 hours, by 73.2% at 48 hours, and 67.0% at 72 hours.

### Effects of TAA on the expressions of the mRNAs of cytokines (Figure 3)

**Figure 3:**
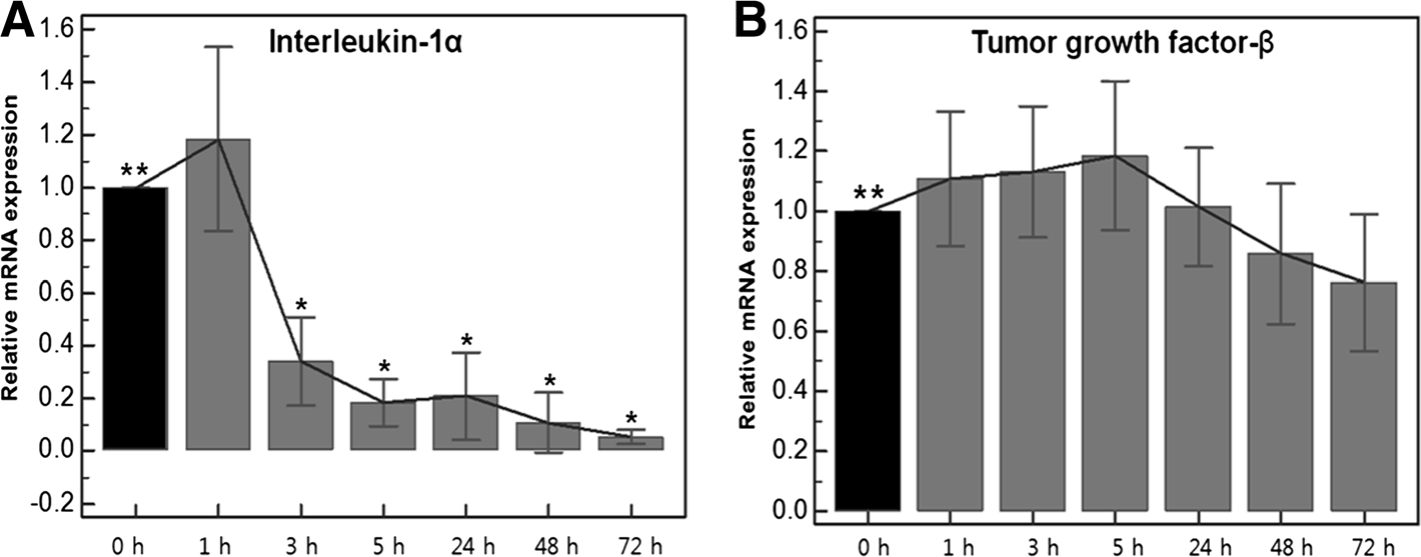
**Results of quantitative real time polymerase chain reaction of cytokine after triamcinolone acetonide treatment (0, 1, 3, 5, 24, 48, 72 hours).** (**A**) Changes in mRNA expressions of interleukin-1α (**B**) Changes in mRNA expressions of tumor growth factor-β **Baseline *Statistically significant values compared to baseline.

#### IL-1Î±

The IL-1α mRNA expressions were significantly affected by TAA at all treatment times (*p* < 0.0001, by repeated measures ANOVA). Its expression was significantly reduced after 3 hours of incubation with TAA (*p* < 0.0001) and this was followed by a further gradual decrease to 72 hours.

#### TGF-Î²

The TGF-β mRNA expressions also differed at the seven treatment times (*p* = 0.0187, by repeated measures ANOVA). However, no values were different from baseline, but it was only differed between the expression at 5 and 72 hours after TAA application (*p =* 0.0341).

### Effects of TAA on tenocyte viability (Figure 4)

**Figure 4:**
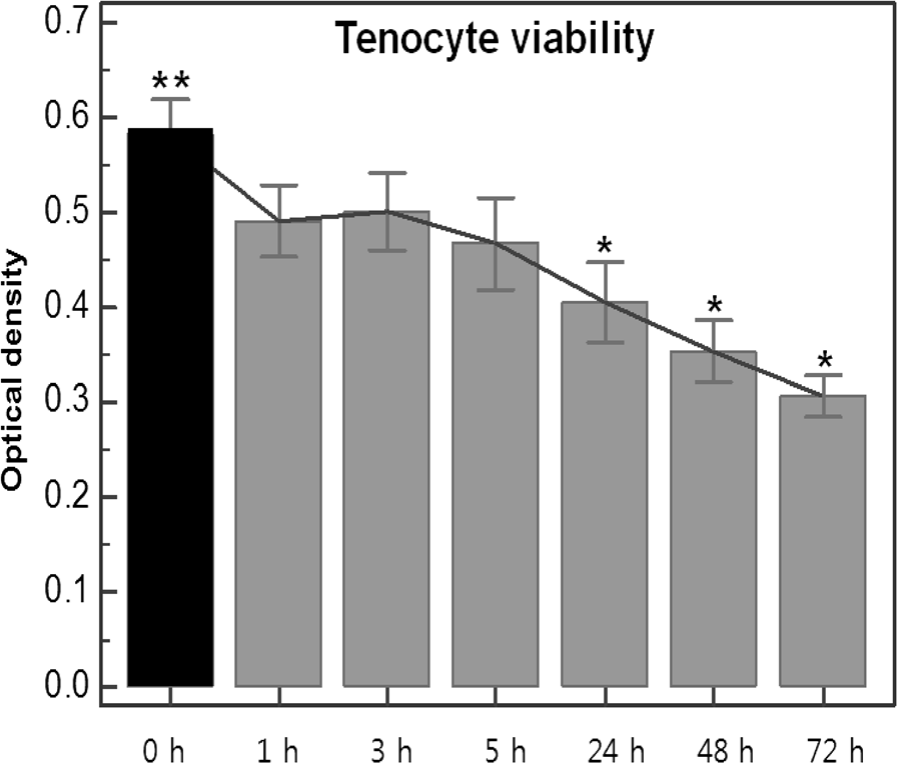
**Changes of tenocyte viability after triamcinolone acetonide treatment (0, 1, 3, 5, 24, 48, 72 hours) by MTT assay.** **Baseline *Statistically significant values compared to baseline

After TAA treatment, the optical densities gradually decreased (*p* < 0.0001, by repeated measures ANOVA). No significant differences were observed between tenocyte viabilities at 0, 1, 3, and 5 hours and at baseline, but at 24, 48 and 72 hours tenocyte viabilities were reduced by 30.4, 39.3, and 47.4%, respectively, versus baseline (*p <* 0.0001, *<* 0.0001, *<* 0.0001, respectively).

### Correlation analysis between the five variables

**Figure 5:**
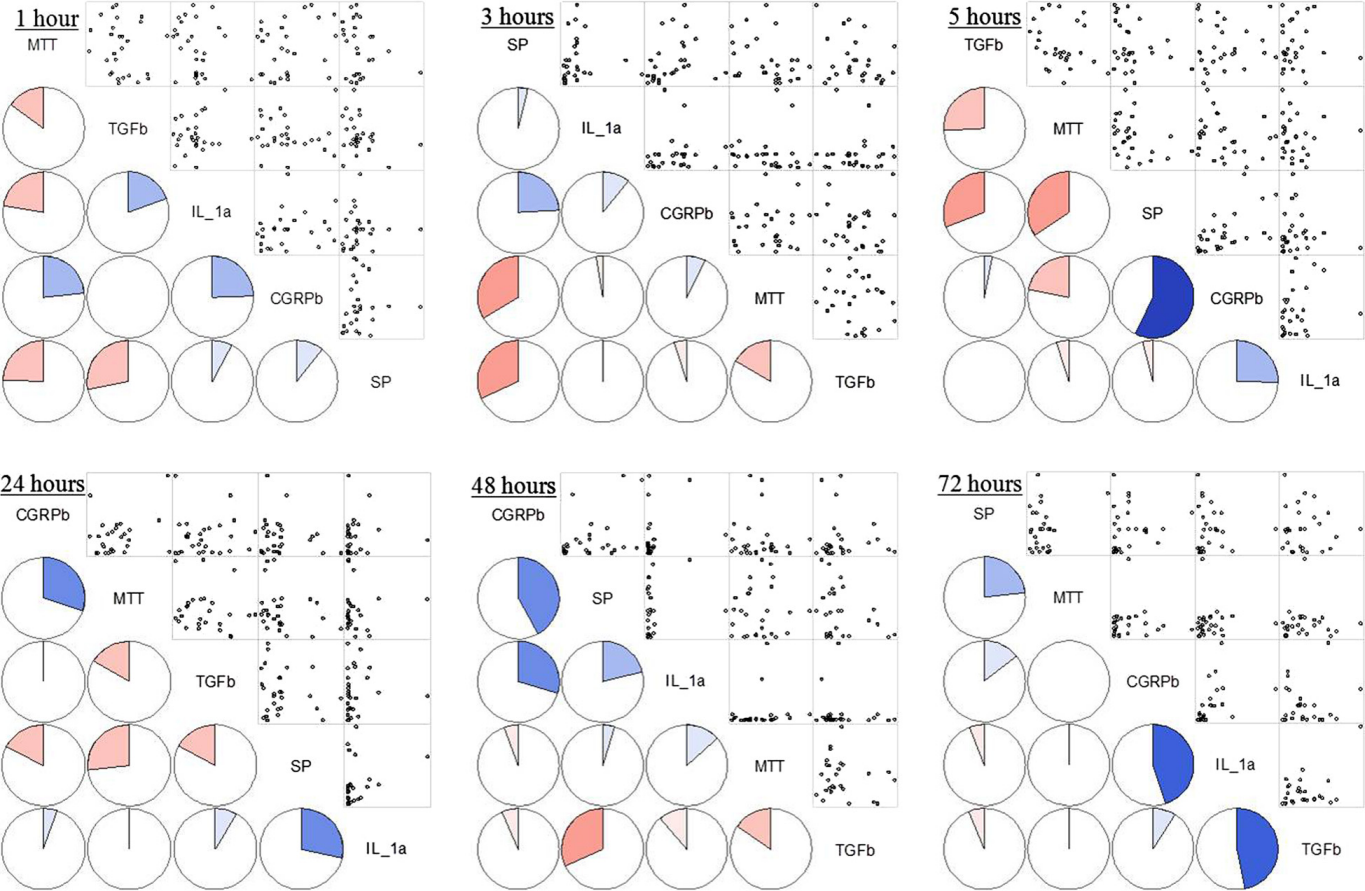
**Pearson’s correlation analysis between between 5 variables at each time.** Analysis reveals no correlation except the CGRP against interleukin-1α after triamcinolone acetonide treatment 72 hours after triamcinolone acetonide treatment. Left lower half: Corrgram shows the correlations by the color and intensity. Right upper half: Typical scatter plot. SP = Substance P, CGRP = Calcitonin gene related peptide, IL-1α = Interleukin-1α, TGF-β = Tumor growth factor-β, MTT = 3-(4,5-dimethylthiazol-2-yl)-2,5-diphenyl-tetrazolium bromide (MTT) assay.

**Figure 6:**
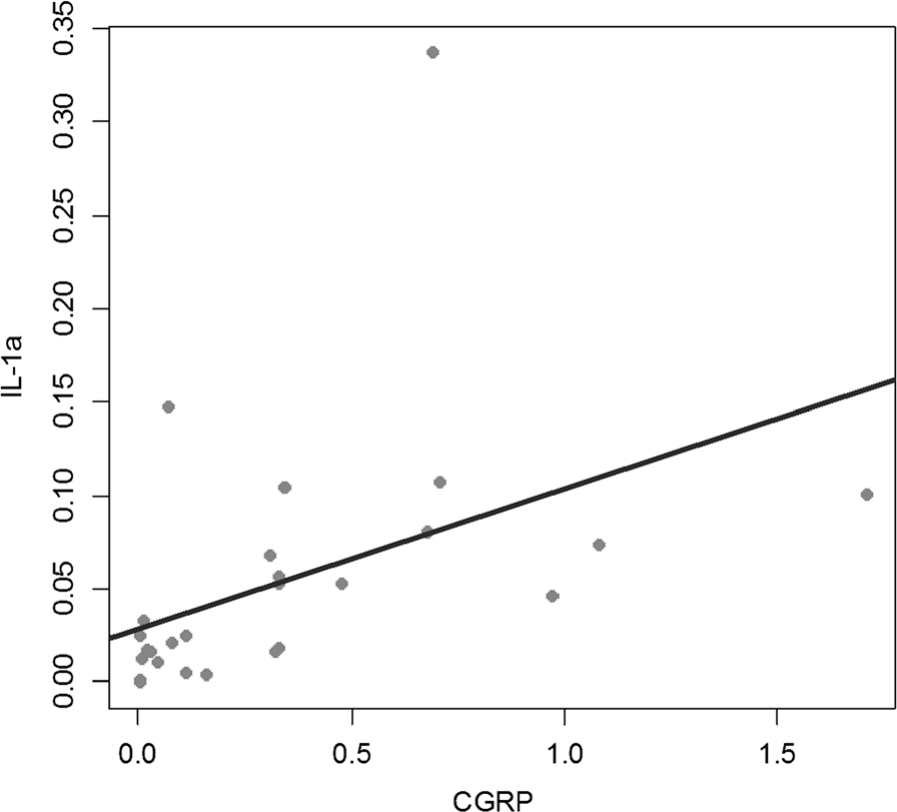
**Correlation plots of mRNA expressions for CGRP against interleukin-1α by Pearson’s correlation analysis at 72 hours after triamcinolone acetonide treatment.** The lines of best fit are indicated on the graphs. CGRP = Calcitonin gene related peptide.

Pearson's correlation analysis at each time point revealed no correlation between the five variables (SP, CGRP, IL-1α, TGF-β, tenocyte viability) after 0, 1, 3, 5, 24, and 48 hours of TAA treatment (Figure 5). However, at 72 hours, a positive correlation was found between the expressions of CGRP and IL-1α (*p* = 0.0184 and *r* = 0.45) (Figure 6).

## Discussion

The present study was undertaken to determine molecular mechanism responsible for the effects of corticosteroid on the neuropeptide and cytokines in lateral epicondylitis. In addition, we examined tenocyte viabilities to investigate the detrimental effects corticosteroid has on tendon integrity.

The previous studies about neurogenic inflammation and lateral epicondylitis have used an immunohistochemical approach. However, immunohistochemical studies have some disadvantages, such as, photobleaching, marker infidelity, and lack of specificity. Furthermore, they rely on qualitative rather than quantitative assessments of effects, and even when results are scored, intra observer variability is problematic. Therefore, in the present study, the mRNA expressions of SP, CGRP, IL-1α, and TGF-β were determined by quantitative real-time PCR.

Before operations, 20 patients were treated with local TAA injection. Five patients were injected once, 7 patients twice, and 8 patients three times. These local injections may have influenced outcomes because corticosteroid infiltration has been reported to cause the necrosis of tendon collagen [17]. However, surgery was performed a minimum of six months after last injections when the patient had sustained or recurred tenderness over the lateral side of the elbow. Thus, it appears reasonable to assume that the effects of previous local injections would have been minimal, as has mentioned previously [8,11].

To decide on the concentration of TAA to use in the present study, we examined the effects of TAA at 25, 50, and 100 mM on tenocytes in small number of cases, and attempted to assess its dose-dependent effect on neuropeptides, cytokines and tenocyte viabilities. However, no dose-dependent effect was observed due to the poor solubility of TAA in culture medium, as described by Herbert et al. [14] Wong et al. [15] reported medical preparations of TAA used for local injections have concentrations over 10 mM, and added that tissue levels immediately after a local glucocorticoid injection would be near that in preparations, and thus, we treated tenocytes with TAA at 100 mM (the same concentration used for injection at outpatient clinics). Furthermore we did not add any solvent except saline. In outpatient department (in vivo), TAA was injected only with normal saline or lidocain.

The time points used in the present study were obtained by reviewing the literature. In several studies, tenocyte viability was analyzed after 2 to 14 days of incubation with corticosteroid [14,15]. However, we considered that neuropeptides and cytokines would response to corticosteroid rapidly because steroid injection in outpatient clinics sometimes improves pain within 24 hours. Accordingly, we performed analysis at 7 time points, at 0 hours (baseline), and after 1, 3, 5, 24, 48, and 72 hours TAA treatment to detect the early effects of corticosteroid.

The peripheral nervous system, acting through neuropeptides, not only relays sensory information to the central nervous system, but also plays an effector role in inflammatory, proliferative, and reparative processes after injury. Of these neuropeptides, SP and CGRP are known to be mainly involved in neurogenic inflammation. Not only do SP and CGRP transmit nociceptive information to the spinal cord, but they are also involved in vasodilation and plasma extravasation [18].

It was previously postulated that the level of SP innervation within tendon tissue is limited. However, recent studies have investigated the expressions of SP and neurokinin-1 receptor in tenocytes, and found that their expression levels are higher in tenocytes in human Achilles tendinosis [19]. In 2010, Lui et al. [20] also reported that the expressions of SP and CGRP are increased in tendon fibroblasts, chondrocyte-like cells, and calcific deposits after collagenase-induced tendon injury. Therefore, we assumed that the cultured tenocyte from lateral epicondylitis has the abundant expression of neuropeptide mRNA in contrast to the normal or unaffected tenocyte which express the neuropeptide mRNA minimally. We also hypothesized that the mRNAs of SP and CGRP might be over-expressed in the tenocytes of lateral epicondylitis in vitro, and preliminarily performed the analysis of mRNA expression from healthy tenocyte, and compared the results with the diseased group. Actually it is not easy to harvest normal healthy tenocyte from human being, consequently the sample number of control was limited to only 5 tendons. However, we found the expressions of neuropeptide and cytokine in control were significantly lower than that of the diseased group (Figure 1). Furthermore several reports have been already demonstrated the increased expression of neuropeptide in the tenocyte of lateral epicondylitis and concluded that the neuropeptide might be the important factor in the pathogenesis of lateral epicondylitis [7,20]. We also did determine the increased expressions of neuropeptides in diseased tenocytes, and we found that the expression of CGRP was significantly inhibited by corticosteroid.

Although the reaction mechanisms responsible for the effects of corticosteroids are not fully understood, it has been reported that corticosteroids can modulate cytokines and other inflammatory mediators [21]. Corticosteroids bind to specific intracellular receptors that function as ligand-induced transcription factors, and thus, modulate the expressions of particular target genes. Furthermore, the expressional levels of various cytokines, such as, IL-1, IL-2, IL-6, IL-8, and interferon-γ are decreased by corticosteroids [22].

Interleukin-1α is an initial phase cytokine responsible for inflammation. We observed that the mRNA expression of IL-1α significantly decreased by 65.8% at 3 hours and then gradually decreased by 94.7% after treatment with TAA for 72 hours. We suppose that these changes are responsible for the symptomatic improvement shown after corticosteroid injection in lateral epicondylitis.

TGF-β is one of the repair-phase cytokines and is involved in the synthesis and deposition of collagen, and scar formation after tendon transection [23]. The effects of corticosteroid on TGF-β are controversial. Some researchers have reported that steroids upregulate TGF-β expression [22,24] but Tempfer et al. [14] reported that TAA inhibits the production of TGF-β by human dermal fibroblasts.

In this study, the TGF-β mRNA expression was slightly higher after treatment for 5 hours without statistical significance, and then started to be inhibited at 24. We hypothesize that these responses are reflective of secondary changes associated with compromised tenocyte viability. However, Pearson's correlation analysis did not reveal any significant correlation.

Steroid injections are also known to decrease neurogenic edema, and to influence the effects and levels of SP and CGRP [8,25]. Ihara et al. [26] studied the effect of glucocorticoids on the regulation of substance P receptor mRNA in rat pancreatic acinar AR42J cells by northern blot analysis using cloned cDNA. It was found that the expression of substance P receptor mRNA was selectively and negatively regulated by glucocorticoid. In the present study, treatment with TAA for 24 and 48 hours significantly reduced the expression of SP mRNA, and subsequently its expression increased slightly. These results suggest that SP is temporarily inhibited by corticosteroid.

On the other hand, CGRP mRNA expression showed a rapid and significant decrease at 24 hours, and this was not recovered. Thus, we speculate that the inhibitionof CGRP is probably a more significant molecular event than the inhibition of SP in reaction mechanism responsible for the effects of corticosteroid in lateral epicondylitis.

In addition to the mRNA expressions of neuropeptides and cytokines, we examined the effect of TAA on tenocyte viability using an MTT assay. In particular, steroids are known to induce adverse histologic changes and reduce the mechanical strengths of tendons [15,27]. In the present study, TAA was found to gradually reduce tenocyte viability by 39.3% at 48 hours and by 47.4% at 72 hours versus baseline.

It could be questioned that the changes of tenocyte viability could affect to the expression of neuropeptide and cytokine. However, statistically the tenocyte viability was decreased significantly 24 hours after TAA treatment. But, although the CGRP was decreased significantly 24 hours after TAA treatment, substance P and interleukin was decreased significantly 1 and 3 hours after TAA treatment (before 24 hours). So we hypothesized that it would be more reasonable that the changes of neuropeptide and cytokine were not affected by the tenocyte viability.

Several studies have addressed the relationship between neuropeptides and cytokines because neuropeptides are probably potent stimulators of proinflammatory cytokine generation [28]. Our study demonstrates a significant positive correlation between CGRP and IL-1α (*p* = 0.0184 and *r* = 0.45) after 72 hours of TAA treatment. These results support the idea that the neuropeptide, CGRP might have effect on the synthesis of IL-1α without inflammatory cell infiltration in this lesion and that neurogenic inflammation underlies the pathogenesis of lateral epicondylitis.

Summarizing, the mRNA expressions of neuropeptides were detected in the lesions of lateral epicondylitis, and TAA treatment was found to inhibit the expressions of the mRNAs of neuropeptides, especially of CGRP, in 24 hours, and the expressions of cytokine mRNAs, especially that of IL-1α, in 3 hours. Furthermore, the expression of SP mRNA was maximally inhibited by TAA at 24 hours and recovered at 72 hours, whereas the expression of TGF-β mRNA was slightly increased versus baseline until 5 hours, and subsequently decreased. In addition, MTT assays showed that tenocyte viability was significantly decreased by TAA at 24 hours, and a significant positive correlation was found between the relative mRNA expressions of CGRP and IL-1α after 72 hours of TAA treatment.

## Conclusions

We postulate that the reaction mechanism of corticosteroid treatment in lateral epicondylitis is associated with the inhibitory effect on not only cytokines but also neuropeptides such as, CGRP. However it should be noted that the tenocyte viability is simultaneously compromised. Furthermore our results revealed that CGRP and IL-1α had significant positive correlation, and this supports that neurogenic inflammation might play a role in the pathogenesis of lateral epicondylitis.

## Abbreviations

(SP): Substance P; (CGRP): Calcitonin gene related peptide; (IL): Interleukin; (TGF): Tumor growth factor; (PCR): Polymerase chain reaction; (MTT): 3-(4,5-dimethylthiazol-2-yl)-2,5-diphenyl-tetrazolium bromide; (PBS): Phosphate-buffered saline; (DMEM): Dulbecco's modified Eagle's medium; (RH): Relative humidity; (TAA): Triamcinolone acetonide; (DEPC): Diethylenepyrocarbonate.

## Competing interests

Each author certifies that he or she has no commercial associations (eg, consultancies, stock ownership, equity interest, patent/licensing arrangements, etc) that might pose a conflict of interest in connection with the submitted article.

## Authors’ contributions

SHH participated in writing and tissue sampling. HJA and JYS participated in gene analysis and MTT assay. DES and YDK participated in analysis of results. JSS participated in writing. SCL participated in study design and writing. All authors read and approved the final manuscript.
